# Artificial Intelligence in the Screening, Diagnosis, and Management of Aortic Stenosis

**DOI:** 10.31083/j.rcm2501031

**Published:** 2024-01-17

**Authors:** Yuxuan Zhang, Moyang Wang, Erli Zhang, Yongjian Wu

**Affiliations:** ^1^Department of Cardiology, State Key Laboratory of Cardiovascular Disease, Fuwai Hospital, National Center for Cardiovascular Diseases, Chinese Academy of Medical Sciences and Peking Union Medical College, 100037 Beijing, China; ^2^Center for Structural Heart Diseases, State Key Laboratory of Cardiovascular Disease, Fuwai Hospital, National Center for Cardiovascular Diseases, Chinese Academy of Medical Sciences and Peking Union Medical College, 100037 Beijing, China

**Keywords:** aortic stenosis, artificial intelligence, screening, risk stratification, TAVR, surveillance

## Abstract

The integration of artificial intelligence (AI) into clinical management of aortic stenosis (AS) has redefined our approach to the assessment and management of this heterogenous valvular heart disease (VHD). While the large-scale early detection of valvular conditions is limited by socioeconomic constraints, AI offers a cost-effective alternative solution for screening by utilizing conventional tools, including electrocardiograms and community-level auscultations, thereby facilitating early detection, prevention, and treatment of AS. Furthermore, AI sheds light on the varied nature of AS, once considered a uniform condition, allowing for more nuanced, data-driven risk assessments and treatment plans. This presents an opportunity to re-evaluate the complexity of AS and to refine treatment using data-driven risk stratification beyond traditional guidelines. AI can be used to support treatment decisions including device selection, procedural techniques, and follow-up surveillance of transcatheter aortic valve replacement (TAVR) in a reproducible manner. While recognizing notable AI achievements, it is important to remember that AI applications in AS still require collaboration with human expertise due to potential limitations such as its susceptibility to bias, and the critical nature of healthcare. This synergy underpins our optimistic view of AI’s promising role in the AS clinical pathway.

## 1. Introduction

Aortic stenosis (AS) is the most prevalent valvular heart disease (VHD) in the western world [1], often manifesting as degenerative or calcific, it is characterized by progressive narrowing of the aortic valve. Without proper intervention, severe AS carries a high risk of mortality [2]. The global impact of AS is escalating, driven by an ageing population and the age-related progression of this condition, as suggested by multiple studies [1, 3, 4, 5]. This underscores the urgent need to improve global management of AS.

Recent breakthroughs in artificial intelligence (AI) from medical fields including cancer biology and genomics [6], have catalyzed enthusiasm for its application to VHD, with a particular emphasis on AS. This is summarized in the Graphical Abstract. Considerable evidence suggests that AI use can enhance the evaluation and management of AS patients at each stage of care. AI facilitates comprehensive screening, spanning age groups from children at risk for congenital VHD or rheumatic fever, to seniors with degenerative AS [7]. Moreover, AI aids in precise diagnosis and improved risk stratification—isolating true high-risk cases among patients labeled with “severe” AS—as well as optimizing treatment options, including pre-procedural evaluations for transcatheter aortic valve replacement (TAVR) [8, 9]. AI achieves these outcomes by synthesizing available patient data including electronic health records (HER), genetic markers, auscultation findings, electrocardiograms (ECG), echocardiograms, and imaging from computed tomography (CT) or cardiovascular magnetic resonance (CMR).

This article provides a comprehensive review of recent advances and emerging concepts of AI application to AS (Table 1, Ref. [7, 10, 11, 12, 13, 14, 15, 16, 17, 18, 19, 20, 21, 22, 23, 24, 25]), with a focus on the integration of AI into pre-clinical and routine clinical management of AS. The final section of this article will address current limitations in AI-AS research methodology, and propose avenues for future research directions for this multifaceted disease with the assistance of AI.

**Table 1.** **Artificial intelligence applications in clinical pathway of aortic stenosis**.

AI in AS	AI techniques	Description	Examples
Screening	Natural language processing	Analyzes and understands human language	- Analysis of ECG and medical history from electronic health records system [10, 11].
	Supervised machine learning	Learns patterns from labeled data	- Integration of patient data for risk score/stratification of AS [16].
Diagnosis &treatment	Computer vision	Utilizes visual data for analysis	- Automated echocardiogram analysis for valvular disease diagnosis [12].
	Image segmentation	Identifies and outlines structures in images	- Generate 3D reconstruction of aortic root based on CT [13, 14, 21, 22] and echocardiography [7, 15, 20, 23] or other imaging modalities.
	Unsupervised machine learning	Discovers patterns without labeled data	- Phenotyping studies based on data patterns [17, 18, 19].
	Computational fluid dynamics	Simulates fluid behavior for interventions	- Simulation for transcatheter aortic valve replacement [24, 25].

AI, artificial intelligence; AS, aortic stenosis; ECG, electrocardiograms; 3D, 3-dimensional; CT, computed tomography.

## 2. Artificial Intelligence in the Clinical Pathway Workflow for Aortic Stenosis

### 2.1 Overview

The application of AI for AS has attracted significant attention, particularly in relation to optimizing echocardiographic assessments. AI application has enabled the full automation of primary AS evaluation [12, 26]. AI algorithms successfully merge echocardiographic data with pertinent clinical information, allowing for the identification of distinct sub-phenotypes among patients with clinically severe AS, a task difficult to achieve through conventional statistical methods or current AS knowledge [17, 27]. Another potential application in AS management that has been met with considerable enthusiasm is the use of AI to assist or automate the planning process for TAVR [24, 28]. This review will be structured chronologically, with a comprehensive examination of AI for screening, diagnosis, and treatment (mainly TAVR) within the general context of AS management (Graphical Abstract).

### 2.2 Massive Screening Made Possible: Primary Prevention

While AS is often fatal once symptoms develop, most AS patients remain under-diagnosed until the late stage [2, 29]. Prior to the onset of symptoms, patients undergo a prolonged subclinical period defined as aortic sclerosis [30]. Early detection is critical, as timely intervention significantly improves the prognosis and outcomes in patients experiencing chronic AS onset [31]. Healthcare and budgetary limits restrict the ability of current clinical diagnostic tools such as echocardiograms to provide large-scale screening in high-risk populations [32, 33]. However, a newly-developed and wearable ultrasound imager [34] enables continuous, real-time cardiac assessments, highlighting the benefit of adopting novel technologies from other fields.

ECG technology is likely to be among the first medical instruments to adopt AI, starting with rule-based clinical decision-making [35, 36]. The interpretation of ECG has evolved thanks to exciting developments in computer vision (CV) and associated technologies, including signals processing and wavelet analysis [37, 38]. AI-assisted ECG interpretation has already made substantial progress in other domains of cardiology, showing excellent performance for the detection and classification of arrhythmia [39], ST changes [40] and additional cardiac abnormalities. The recent development of rECHOmmend, an ECG-based screening tool from Ulloa-Cerna *et al*. [41] offers a new option for population screening. The machine learning system integrates clinical factors and laboratory measurements with structured ECG data to simulate physician decision-making. It identifies patients who are at high risk of structural heart disease, flagging them for further ECG evaluation. Validation studies have confirmed the accuracy and reliability of clinical referrals made by rECHOmmend [41]. Elias *et al*. [42] reported an alternative deep learning prediction model trained exclusively on ECG figures and focused on the screening of left-sided VHD. Another study revealed that individuals flagged as “false-positives” by AI had double the risk of developing moderate or severe AS during 15-year period compared to the “true-negative” population [8]. This demonstrates the possibility of using AI-ECG to predict the onset and progression of AS.

The generalizability of AI models improves with increased amounts of training data [43, 44]. The “federated learning” technique involves training of the model with multiple datasets acquired from different institutions without data merging. This has significantly enhanced the performance of unseen datasets [43]. In conclusion, AI-empowered ECG interpretation shows considerable promise for the screening of undiagnosed AS patients in an aging society.

Another possible AI-assisted screening tool for AS is AI-auscultation. In recent decades, the dependency on auscultation has declined due to advances in cardiac imaging and physician proficiency with this technique [8]. Furthermore, less than half of patients with moderate or severe AS exhibit systolic murmurs [45]. Nevertheless, auscultation remains widespread due to its portability and cost-effectiveness [46], making it suitable for application in non-clinical settings such as community screening. The acquisition of heart sounds for AI models is comprised of two major components: the digital stethoscope, and the phonocardiogram (PCG), which visualizes the waveform of heart acoustics [47]. Over the years, substantial effort has been made to classify different heart sounds into normal or abnormal groups [48, 49]. More recent studies have attempted to discriminate between different valvular conditions by recognizing specific murmurs, such as the systolic murmurs of AS [50, 51, 52, 53]. In a noteworthy animal experiment, Dargam *et al*. [54] developed an ensemble-learning-based algorithm that uses S2 sounds extracted from PCGs to predict aortic valve calcification. This has major clinical significance due to the poor prognosis of patients with calcified AS [55]. The encouraging progress of AI-empowered auscultation also has potential as a screening modality for patients with severe AS, especially in the community setting.

The integration and utilization of reports such as ECG, echocardiography, and CMR stored in Electronic Health Record (EHR) systems presents a significant challenge due to the unstructured nature of these records [56]. Natural Language Processing (NLP) is an integrated algorithm that enables computers to comprehend texts and speeches [57, 58]. NLP holds promise for identifying AS patients and extracting relevant clinical information from large, non-organized EHR databases [10, 11, 59, 60]. Despite this promise, concerns remain regarding the accuracy, validity, and applicability of applying NLP models across datasets from different institutions [61]. Although NLP can effectively learn nuanced linguistic expressions from diverse document structures at one facility, the model might not correctly interpret data from other institutions [62]. However, recent studies indicate some success in transferring NLP models between multiple facilities, alleviating concerns over their portability [59, 63, 64, 65]. The application of NLP to EHR systems represents a considerable advance in facilitating more effective population management on a larger scale.

In addition, simultaneous screening for AS during examination for other conditions such as lung low-dose CT and CT for breast cancer is also possible, thus providing even greater scope for AS screening [66, 67, 68]. By exploiting all available pre-clinical resources including multimodal imaging, clinical results and biochemical data, AI may facilitate patient referral for advanced examination in a non-invasive and cost-effective manner. Not only can it overcome limitations in expertise and human error by ensuring consistency of advice, it can also allow large cohorts to have rapid interaction with physicians.

### 2.3 Bridging of Image Assessment with Diagnosis

In the clinical setting, AS diagnosis relies primarily on patient symptoms and severity, as indicated by imaging assessments including valvular stenosis and the function of up-stream or down-stream structures. Advances in AI, particularly within the field of CV, have allowed significant progress in bridging image assessment with clinical diagnosis of the disease. Two notable studies have developed fully automated workflows for AS diagnosis using color Doppler ECG images [12] and videos [26]. The process begins with view identification, progresses through structure segmentation and measurement, quantification, and culminates with disease classification [12]. Utilizing this workflow, Yang *et al*. [26] developed a diagnostic tool for VHD that achieved an impressive area under the curve (AUC) value of 0.97 for AS diagnosis. Additionally, the AI-driven echocardiography model provided precise predictions for the accurate peak aortic jet velocity and transvalvular mean pressure gradient [26]. While view identification allows AI to quickly identify key images suggesting valvular abnormalities [9], existing research has largely concentrated on other structural heart diseases such as mitral and tricuspid regurgitation [69, 70, 71], rather than AS.

Automated provides precise outlines of key anatomical features such as the aortic root and left ventricle from various imaging modalities. This forms the basis for subsequent AI analysis leading to personalized simulations that aid in treatment planning and outcome prediction. Over the past decade, considerable efforts have been made to segment and reconstruct the aortic root using various imaging modalities in order to approach the “ground truth” [13, 14, 72, 73], which in most cases is the underlying anatomical structure manually delineated by clinicians. Using unlabeled MRI sequences, Fries *et al*. [74] identified malformed aortic valves and linked specific malformations to elevated risk of future cardiac events. Segmentation of the aortic valve and detection of anatomic malformations are made possible in real-time by echocardiography [15]. Bhuva *et al*. [75] developed an AI model to retroactively analyze the segmented left ventricle (LV) following aortic valve replacement (AVR). Using CMR data from 116 symptomatic AS patients, the AI model exhibited superior sensitivity in detecting regional variations in LV wall thickness when compared to traditional methods, a finding corroborated by Duffy *et al*. [76].

Automatic analysis of the AS condition encompasses both morphological and functional assessments. Morphological assessments, often used for pre-TAVR evaluation, leverage neural networks to analyze the volume and Agatston score of aortic root calcifications based on segmentations [77]. These assessments are viable even in low-dose CT for lung screening [68] and auscultation [54]. supervised AI models can also predict the aortic valve area that supports prosthesis sizing, thereby minimizing inter-observer variability [16]. Functional assessment of AS usually refers to valvular hemodynamics and extra-aortic valve cardiac damage, including left ventricular ejection fraction (LVEF) [78, 79, 80] and 4-dimensional flow quantification [81, 82]. These AI-empowered automations are now bridging AS assessment with clinical diagnosis under the current guidelines and clinical criteria [32].

**Fig. 1.**
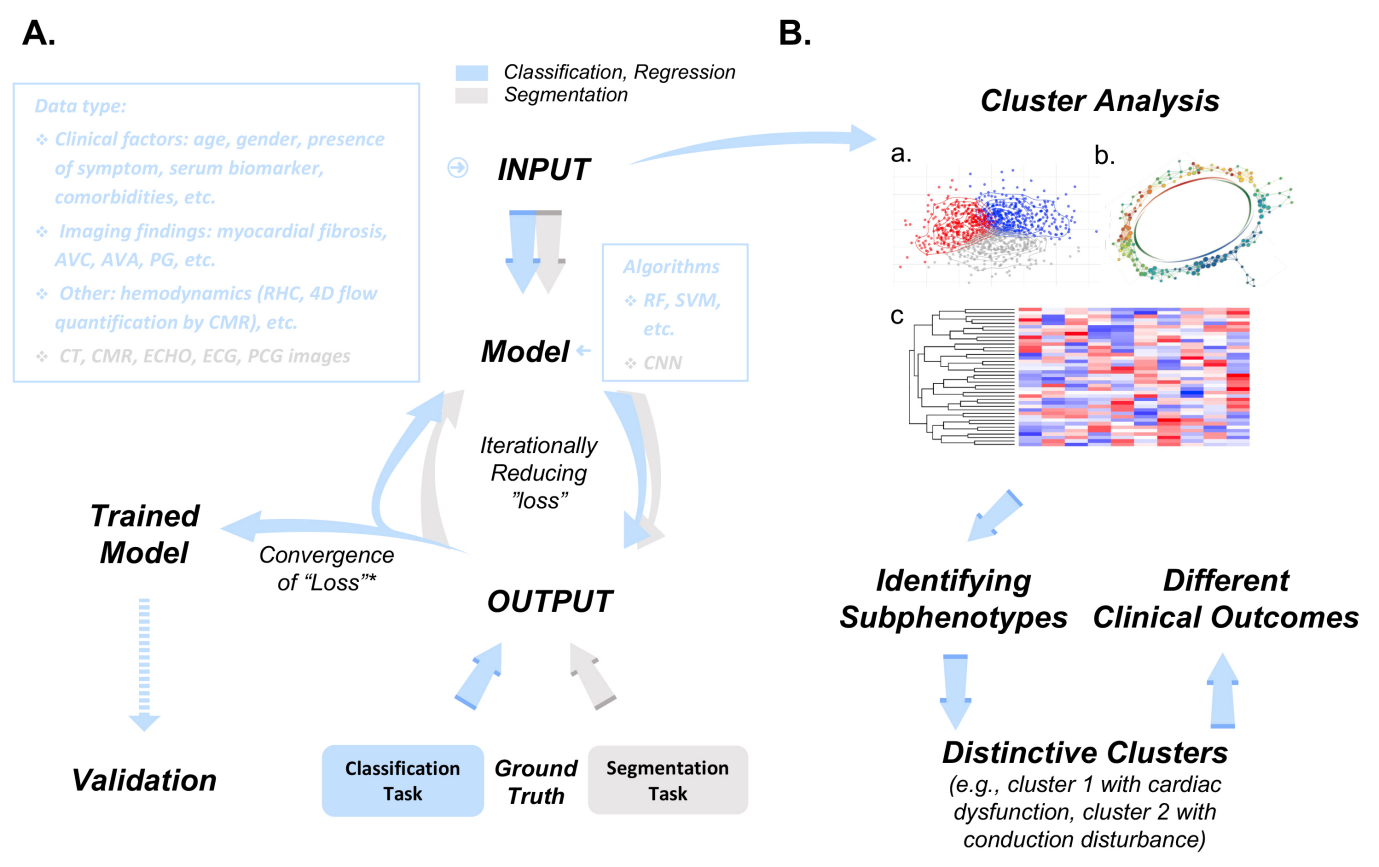
**Methodology of supervised and unsupervised learning in clinical AS assessment and management**. (A) Workflow illustration of supervised (classification/regression) and unsupervised (segmentation) learning tasks in the context of AS assessment and management. Each step in the workflow varies based on the learning task. Input data, representing training material for the model, is used for learning. Classification/regression tasks involve algorithms like random forest and support vector machine, while segmentation tasks often employ convolutional neural networks (CNNs) for encode-decode frameworks. Model output is compared iteratively to the “ground truth”, represented either by labels/categories or annotations, depending on the task. Loss function convergence is achieved by minimizing the gap between output and ground truth. The AS severity grading scheme [30] serves as ground truth for classification/regression, while manual delineations in images such as CT scans [83] serve as gold standards for image segmentation. (B) Unsupervised learning, exemplified by cluster analysis, utilizes similar input data. Algorithms include model-based clustering (a) [18], topological data analysis (b) [19], and agglomerative hierarchical clustering (c) [17], with results visualized using heatmaps and dendrograms. Both supervised and unsupervised models undergo internal and external validation processes. * Convergence of Loss Function refers to the point where the loss can no longer be reduced under current training settings. AVC, aortic valve calcium; AVA, aortic valve area; PG, peak gradient; RHC, right heart catheterization; 4D, 4 dimensional; CT, computed tomography; CMR, cardiovascular magnetic resonance; ECHO, echocardiography; ECG, electrocardiography; PCG, phonocardiogram; RF, random forest; SVM, support vector machine; CNN, convolutional neural network; AS, aortic stenosis.

Despite the workflow proposed by Zhang *et al*. [12], AI-assisted diagnosis of AS is not a logical decision of upstream measurements resembling a clinical decision. Instead of a rule-based diagnostic approach, AI models mostly identify AS through pattern recognition in the features extracted from either imaging or segmentation [16]. This is supervised by pre-defined labels or annotations (e.g., AS or healthy) provided by clinicians (Fig. 1A, Ref. [17, 18, 19, 30, 83]). CV-based AS detection models can also use segmentations derived from other kinds of images, such as continuous waveform recorded by non-invasive, wearable inertial sensors [84], thereby extending the possible application of AI for the assessment of AS conditions. A seamless computational modeling framework based on CV algorithms holds great promise for achieving higher reproducibility in aortic valve analysis, with less intra- and inter-observer variability [81, 85], thus bridging the assessment and diagnosis of AS.

### 2.4 Precise Diagnosis: Insights from Phenotypic Studies

The diagnosis of AS severity is the basis for further decisions regarding treatment. Conventional phenotyping of AS patients has been difficult and limited, due to its dependence on a small number of echocardiography findings including jet velocity, mean gradient, and LVEF [32]. Given the heterogeneity of AS, existing guidelines relying on a limited set of predictors can yield inconsistent assessments of AS severity. This leads to diagnostic ambiguity in cases like low-flow low-gradient AS and borderline AS, resulting in indecision regarding the appropriate treatment. While considerable effort has gone into developing statistical prediction models to delineate AS patient phenotypes and clinical outcomes [86], these models have achieved only modest success in risk stratification. Machine learning (ML) offers a way to overcome this limitation. ML-based models can unearth “hidden” variables within diverse data sources that are not readily identifiable using traditional statistical methods or current guidelines [87]. Both supervised ML and unsupervised cluster analysis are frequently used for this purpose [88] (Fig. 1).

Supervised ML operates by iteratively learning the intricate relationships between input variables and their corresponding outcomes. For instance, a study incorporated 90 clinical variables, LVEF and 57 additional echocardiographic parameters as inputs for supervised ML model [89]. Initially, the model estimates patient outcomes (e.g., survival likelihood) based on randomly assigned weights for each input parameter (e.g., LVEF). The discrepancy between the estimated and actual outcomes, referred to as “loss”, drives the model’s learning process. It refines the weightings of individual variables to minimize this “loss” (Fig. 1A). Over time, the weight for each variable becomes tailored to the learned correlation or causality of the variable with outcome (e.g., LVEF is assumed to be important for AS and so its weighting increases). The ML model can thus provide more accurate prediction of clinical outcome for unseen patients than a statistical prediction model [89]. Several studies have tried to achieve risk stratification of AS by applying different ML algorithms, either through the prediction of clinical outcomes such as referral for AVR [90], cardiac events [91], or mortality [16, 89, 90, 92]. Through this process several outcome predictors have been identified [89, 91, 92, 93].

Unlike supervised ML, unsupervised ML represented by cluster analysis operates without the need for predefined labels or annotations. Unsupervised clustering methods have revealed meaningful phenotypes within AS patient groups based on similarities rather than differences, and without the use of pre-defined diagnostic criteria or assumptions (Fig. 1B). Lachmann *et al*. [17] used unsupervised cluster analysis to group patients according to similarities in their baseline characteristics. These features were obtained from patients undergoing right heart catheterization and echocardiography prior to TAVR, irrespective of the severity of their clinically diagnosed AS. Each cluster showed distinctive clinical characteristics. For example, patients in cluster 3 displayed left and right heart dysfunction with pulmonary hypertension. In contrast, cluster 1 patients presented with regular cardiac function. The unsupervised ML model generated strong outcome predictions, with 2-year survival rates after TAVR of 90.6% for cluster 1 and 77.3% for cluster 2 [17]. Lachmann *et al*. [94] found that the discriminative power of cluster analysis was based on identifying the inherent yet obscure irreversibility of cardiac dysfunction (and therefore poor prognosis), rather than the obvious characteristics at baseline. further studies support the use of risk stratification using unsupervised cluster analysis [ 18, 19, 27, 95, 96]. In addition to clinical outcomes, Sengupta *et al*. [19] confirmed the reliability of this methodology by using CT and CMR to assess the severity of AS in different clusters. Patients who clustered in the severe phenotype had a higher aortic valve calcium score, greater ventricular mass, and more cardiac fibrosis [19]. It is worth noting that the high-severity phenotype identified by cluster analysis is often associated with cardiac dysfunction and other structural abnormalities [17, 18, 19, 27, 95, 96]. Together, these AI-generated results highlight that AS is more of a “myocardial continuum” with sequential upstream damage, rather than an isolated structural condition. The novel phenotyping of AS by supervised prediction and unsupervised clustering has allowed the issue of AS severity to be addressed. Risk stratification for discordant AS patients can now be refined on the basis of a valve-myocardium functional continuum.

### 2.5 “Intelligent” TAVR: Decoding-Encoding Framework

TAVR has gained traction as a treatment for severe AS, extending its application from high-risk surgical candidates to those at low-risk [97]. AI offers advantages across the entire TAVR treatment process, including diagnosis, treatment and prognosis. Most AI-assisted TAVR models are currently built using different imaging modalities, including ECG [20, 98], CT [21, 22, 72] or cardiac magnetic resonance imaging (MRI) [82, 99, 100]. Segmentation is a foundation for many models utilizing a so-called “decoding-encoding” framework. In this setting, the information contained within images is first extracted as “features” in an abstract way that is understood by computers (decoding). These features are subsequently reorganized and then reconstructed by gradually approaching the “ground truth” (encoding). The “decoding-encoding” framework is thus deeply embedded in the planning for “intelligent” TAVR.

CT is the most well-established imaging modality for the pre-procedural evaluation of TAVR [101, 102]. The precision and reproducibility of CT-derived aortic valvular dimensions are therefore of paramount value, since they determine the downstream workflow including prothesis selection, prediction of outcome, and TAVR simulation. Manual measurement of valvular dimensions is often semi-automated and shows high repeatability [103, 104]. However, manual measurement is time-consuming, inefficient, and requires multiple readers to guarantee precision [103], hence the need to automate the evaluation of patients referred for TAVR.

Extensive efforts have been undertaken to automate pre-TAVR valvular evaluations, particularly by measuring aortic annular planimetry [ 7, 13]. The process begins by identifying the aortic valve [13, 82, 105, 106, 107], and is often referred to as landmark localization, or annular plane detection. Other methods that exploit advances in the field of CV include segmentation [21, 22, 72, 99, 100] and automatic measurement of the reconstructed geometry of the aortic root [20, 23]. Automatic analysis software based on ECG rather than CT also has potential for automating the measurement of aortic annular planimetry. This was demonstrated with the remarkable agreement between CT-derived results [7, 98, 108], represented by Aortic Valve Navigator from Philips [108], and eSie from Siemens [7].

Dimensional analysis of the annular geometry is crucial for selecting the apporpriate transcatheter heart valve (THV). AI-driven THV sizing has proven reliable, as observed by the excellent agreement between human experts and AI models [23, 98, 108, 109, 110]. In 2019, Astudillo *et al*. [28] demonstrated that an AI model can swiftly and accurately personalize prostheses size based on automated CT annular measurements. This rule-based approach uses automated measurements to inform the selection. The final THV type is determined by two parameters, the perimeter for the Self-Expandable Valve and the area for the Balloon-Expandable Valve [28]. However, this clearly over-simplified the problem of prothesis selection based on an exclusive parameter in a singular dimension, given the complexity of the operative area of TAVR. Attempts have been made to address this dilemma by incorporating all relevant parameters assessed during pre-operative evaluation (including raphe length, calcium burden and calcium distribution) into a THV selection model for the bicuspid aortic valve (BAV). This improved TAVR performance [111], demonstrating the applicability for a more sophisticated selection algorithm. Indeed, the selection methodology for THV should include multiple variables, which is clearly within the scope of AI despite being disregarded in this specific use case. These results suggest that optimized selection of THV would be more “intelligent” by embracing the power of AI.

In addition to recommendations regarding THV sizing, AI could also help guide intra-procedural operations during TAVR. For example, the advent of real-time segmentation of THV and delivery systems based on intra-procedural angiography provides broader views that greatly reduce operational difficulties [107, 112]. Furthermore, procedural techniques such as implantation depth, which is related to peri-operative conduction abnormality [113], could become more refined using patient-specific computer simulation (PSCS) [24, 25]. The current workflow for PSCS can be established through either a finite element and computational fluid dynamics [24, 25, 114], or with tissue-mimicking metamaterial 3D printing [115, 116]. Both methods are based on the reconstructed aortic valve model generated from deep learning methods. By exploiting advances in computational power, PSCS can assess potential interactions between the device and host, thus streamlining the TAVR process. This includes pre-procedural THV selection, guiding the procedural operation, and predicting peri-operative complications based on specific patient characteristics and prior procedural decisions (e.g., implantation depth). Dowling *et al*. [114] used PSCS to retrospectively analyze pre-procedural multi-detector computed topography (MDCT) results from 37 patients with BAV who underwent TAVR. The model accurately predicted THV frame deformation, paravalvular regurgitation, and conduction disturbance after TAVR. The same PSCS system prospectively guided the clinical decisions for 9 patients with BAV referred for TAVR [25]. This resulted in 3 referrals for surgery, and alterations in the size and depth of implantation for THV in 5 patients. In the remaining patient, the simulation predicted a conduction disturbance and implantation of a pacemaker before TAVR was suggested. Due to its of individual clinical characteristic analysis, PSCS holds great promise in predicting TAVR complications, facilitating TAVR recommendations tailored for each patient. Furthermore, the algorithm provides a framework for THV design [117] and possible surveillance of upcoming THV degradation [118], although further evidence is needed to substantiate the latter. Other major peri-operative post-TAVR complications that are predictable from AI models include bleeding [119, 120], permanent pacemaker implantation [121], and early cerebrovascular events [122].

Building on preliminary AI success in predicting post-TAVR complications, many studies have explored AI predictions for TAVR patient long-term outcomes. A recent study by Kwak *et al*. [92] demonstrated the “random forest” ML model could identify CMR markers that independently predict mortality risk in AS patients following aortic valve replacement (AVR). These included late gadolinium enhancement (LGE) >2%, extracellular volume fraction >27%, both large (LVEDVi >80 ${\mathrm{mL/m}}^{2}$) and small (LVEDVi ≤55 ${\mathrm{mL/m}}^{2}$) ventricles, and high (>80%) and low (≤50%) right ventricular ejection fraction [92]. Emerging ML-based models continue to reveal details describing the underlying mechanism of AS [92] enabling the prediction of both intra-hospital [123] and long-term TAVR clinical outcomes [124, 125, 126]. Additionally, 3D AI models have heightened sensitive to nuanced shifts in both global and regional myocardial plasticity, namely LV remodeling before and after TAVR and which has important prognostic value [75]. Nonetheless, the advent of AI-empowered CT fractional flow reserve has enabled the prediction of adverse clinical outcomes in TAVR patients with concomitant coronary heart disease [127, 128]. In tandem with the development of AI, risk stratification models for AS patients undergoing TAVR are expected to significantly improve in the future. This should in turn facilitate and refine AS patient management. Taken together, the emerging evidence has prompted further integration of AI into current TAVR planning, procedures, and long-term management.

## 3. Limitations and Challenges

Despite being an advanced clinical decision support tool, significant questions have been raised about the application of AI in the AS clinical pathway. The major limitation of AI that hinders its widespread application in AS management is that the models depend heavily on the quantity and quality of data. This limitation makes AI models susceptible to the same flaws that characterize traditional statistical methods, especially in the field of clinical medicine. First, echocardiography, the most common form of imaging data in AS patients, has lower resolution and a higher requirement for expertise compared to other imaging modalities. This can introduce bias into the AI models because of “noise” (e.g., artifacts, inter- and intra-observer variability). For example, a prediction model could be trained on a dataset of echocardiography images suggesting AS, but if most of the images are marred by speckle noise (often due to inadequate expertise), a limited number of images may reflect reduced AVA. The deeply flawed dataset would inevitably produce a heavily biased model that is prone to identify AS-echocardiography based on speckle noise rather than on true pathology, such as reduced AVA. Fortunately, the increasing demand for CT dictated by the expansion of TAVI has led to additional resources for AI-AS research with higher resolution than echocardiography. Second, when presented with an imbalanced dataset containing skewed data, irrespective of the data quality [129], the AI models will also be heavily biased towards “noise” due to the distribution of the training data, i.e., patient selection. Third, existing regulations to protect patient privacy also limit data exchange [130], thus current AI research in clinical settings is limited to local patient data from a single institution, and therefore lacks generalizability. For example, CMR images from different hospitals are usually produced using different types of machines and under different settings. The resulting differences in the images present as “noise” to the AI models and blur the essential data, thus leading to a systematic dataset shift [131]. This could explain why NLP models that are well-trained in one EHR system perform worse in another [62]. Hence there is a clear need to mitigate the problem caused by restrictions on data-sharing between health centers and institutions, possibly by using newly developed federated learning systems that do not rely on data sharing [125]. The availability of sufficient qualified data from AS patients will be resolved in future by the increasing use of other imaging modalities with better resolution, together with the introduction of other techniques in computer science such as federated learning and adversarial examples. However, the incorporation of larger datasets is still restricted by computational power, since the processing of more data requires exponentially increased operations, especially for tasks involving CV (e.g., segmentation).

Another problem is the inability to interpret AI models due to the abstraction of features, such as the contour of the aortic root in CV tasks. This creates a “black box” phenomenon making it difficult to assess model bias, while also failing to provide a statistically convincing pathophysiologic explanation for the associations or causality. However, continued attempts have been made to remove “black box” ambiguity by providing human-explainable features [132, 133] and by mimicking the attention mechanism of human vision (i.e., transformer) [134]. These may shed light on the enigmatic yet exciting journey of human intelligence being able to comprehend AI.

Furthermore, it is important to acknowledge the inherent operator-dependence of echocardiography. AI models based on such data must account for variations introduced by different operators, which could impact the accuracy and generalizability of these models [135]. This operator-dependent variability poses a challenge in ensuring consistent and reliable AI predictions across different healthcare settings. Strategies to address this limitation could involve the incorporation of operator-specific factors into the training data, or the introduction of normalization techniques to account for operator introduced variability from different echocardiography practitioners [83]. Furthermore, ongoing efforts to create standardized acquisition protocols could help to mitigate operator-related discrepancies and improve the reliability of AI models that use echocardiographic data. Nevertheless, the moderate performance of AI-based decision systems highlights the need for cautious adoption [136]. While AI can contribute to AS clinical pathways and its applications show promise for expansion, human oversight for the interpretation of findings remains essential given the acknowledged limitations.

## 4. Future Perspectives

In summary, the integration of AI into AS management holds great potential, but also introduces several challenges that require strategic solutions. Collaborative initiatives including multi-center partnerships and federated learning can enhance the representative datasets with greater quality and diversity of data, thereby improving the accuracy and impartiality of AI models. Considering the operator-dependent nature of echocardiography, future AI models should be designed to minimize the impact of variability between different practitioners. The inclusion of operator-specific features and of normalization techniques should ensure consistent and dependable AI predictions.

Furthermore, the use of techniques that give transparent and explainable predictions can increase the clinicians’ confidence in AI-assisted decisions. In view of the importance of the “interpretability” of AI models, this should facilitate their seamless integration into clinical workflows. Validation across a wide array of patient populations is critical for confirming the clinical efficacy of AI tools. Collaborative endeavors involving clinicians, AI researchers, and regulatory bodies can establish rigorous validation protocols to ensure the implementation of AI is both safe and effective. A collaborative approach that also combines human expertise with AI capabilities can yield optimal results. AI can assist clinicians with risk stratification, thus enabling personalized treatment strategies and interventions. While AI can provide predictions, human oversight and interpretation of the predicted results remains indispensable for validation and for ethical considerations. This is particularly important when applying AI models in clinical studies that involve small and specific patient populations.

Addressing equity concerns during the application of AI is complex but imperative. Initiatives that focus on equitable data collection, algorithm development, and AI deployment can mitigate bias and ensure equitable access to accurate diagnosis and treatment. In summary, future applications of AI for the management of AS appear promising. By meeting challenges head-on and fostering collaboration, we can look forward to an era where AI enriches clinical decision-making, improves patient outcome, and revolutionizes the management of AS.

## 5. Conclusions

This review has highlighted recent applications of AI for the assessment and management of AS. AI shows promise not only for early detection of the valvular condition and accurate diagnosis, but also for appropriate referral and treatment decisions of AS patients. However, it is important to recognize the strength AI depends on the data utilized for model development, thus making AI vulnerable to bias. Although AI has many possible applications, the realization of its full potential requires the collaboration of human and machine, especially within the complex context of AS. Further studies into the potential of AI and its synergistic applications for improving the screening, diagnosis and management of AS are therefore warranted.
